# Serum metabolic signatures for acute pulmonary embolism identified by untargeted metabolomics

**DOI:** 10.3389/fmed.2023.1169038

**Published:** 2023-06-02

**Authors:** Ming Xie, Yu Liu, Hui Zheng, Xiaoli Gao, Ran Liu

**Affiliations:** ^1^North China Petroleum Bureau General Hospital, Renqiu, China; ^2^Key Laboratory of Environmental Medicine Engineering, Ministry of Education, School of Public Health, Southeast University, Nanjing, China; ^3^Binjiang District Center for Disease Control and Prevention, Hangzhou, China

**Keywords:** acute pulmonary embolism, diagnosis, metabolites, biomarkers, machine-learning, LASSO

## Abstract

**Background and aims:**

The important metabolic features of acute pulmonary embolism (APE) risk stratification and their underlying biological basis remain elusive. Our study aims to develop early diagnostic models and classification models by analyzing the plasma metabolic profile of patients with APE.

**Materials and methods:**

Serum samples were collected from 68 subjects, including 19 patients with confirmed APE, 35 patients with confirmed NSTEMI, and 14 healthy individuals. A comprehensive metabolic assessment was performed using ultra-performance liquid chromatography-mass spectrometry based on an untargeted metabolomics approach. In addition, an integrated machine learning strategy based on LASSO and logistic regression was used for feature selection and model building.

**Results:**

The metabolic profiles of patients with acute pulmonary embolism and NSTEMI is significantly altered relative to that of healthy individuals. KEGG pathway enrichment analysis revealed differential metabolites between acute pulmonary embolism and healthy individuals mainly involving glycerophosphate shuttle, riboflavin metabolism, and glycerolipid metabolism. A panel of biomarkers was defined to distinguish acute pulmonary embolism, NSTEMI, and healthy individuals with an area under the receiver operating characteristic curve exceeding 0.9 and higher than that of D-dimers.

**Conclusion:**

This study contributes to a better understanding of the pathogenesis of APE and facilitates the discovery of new therapeutic targets. The metabolite panel can be used as a potential non-invasive diagnostic and risk stratification tool for APE.

## Introduction

Pulmonary embolism (PE) is a disease or clinical syndrome caused by the blockage of a pulmonary artery or its branches by various endogenous or exogenous emboli, such as blood clots, air, fat, amniotic fluid, etc. (1). As a result of pulmonary artery occlusion, it can lead to life-threatening but potentially reversible pulmonary hypertension (PH) and right ventricular failure (2). The morbidity and mortality rate of PE is high, not only in China (0.1%) but also in Europe and the USA (0.05%) (3, 4). Early detection of the disease, together with prompt treatment, is considered to be an effective means of reducing mortality (5).

The current ‘gold standard’ for the diagnosis of PE is computed tomography pulmonary artery imaging (CTPA) (6). However, it has limitations due to the adverse effects of contrast agents and radiation damage, which may exacerbate the patient’s symptoms. The most commonly used biomarker is D-dimer (D-D), which is a degradation product of cross-linked fibrin and has shown good sensitivity in the diagnosis of thrombosis, but unfortunately, it does not confirm that a patient has APE, as elevated D-D levels can occur in many conditions such as infection, cancer, and inflammation (7, 8). In addition, the clinical symptoms of APE are similar to those of non-ST-segment elevation myocardial infarction (NSTEMI), which poses a major challenge in the differential diagnosis and subsequent treatment of the two diseases (9, 10). Thus, it is necessary to provide more options for efficient and accurate aid to diagnosis of APE.

In recent years, blood metabolites have received widespread attention as potential biomarkers and have become an emerging area of APE research (11, 12). Existing metabolomic studies of APE have shown that the pathogenesis of APE is closely linked to abnormalities in energy metabolism, such as dramatic changes in the metabolome involved in glycolysis and the tricarboxylic acid (TCA) cycle during APE (13). Metabolomics can monitor hundreds to thousands of metabolites and may help identify significant differences in serum metabolites between APE and NSTEMI.

The purpose of this study was to describe the profile of the serum metabolic profile and to compare the differences between APE, NSTEMI, and healthy individuals using a metabolomics approach. In addition, to explore novel biomarkers of APE, diagnostic and discriminant models of APE were developed using classifiers based on the Least Absolute Shrinkage and Selection Operator (LASSO) and logistic regression algorithms. The study also aimed to explore the potential biological functions and metabolic pathways of different metabolites, providing clues to novel diagnostic, preventive, and therapeutic strategies for APE.

## Materials and methods

### Subjects

Sixty-eight subjects, including 19 patients with confirmed APE (14, 15), 35 with confirmed NSTEMI (16), and 14 healthy individuals, were recruited consecutively from the Huabei Petroleum Administration General Hospital. Ethical approval was obtained for the study, and all participants gave informed consent. All participants had complete clinical information and underwent basic clinical investigations, including routine blood tests, biochemistry, and D-Dimer. All patients with APE were identified by CTPA and met the guidelines for diagnosing and managing acute pulmonary embolism (14, 15). Healthy candidates were recruited from a population of people who had a medical examination which showed that they were healthy and did not have any respiratory or cardiovascular disease. Patients with APE, healthy individuals, and patients with NSTEMI were matched for age and sex. Serum samples were collected from patients with APE at the onset of the disease (pre-treatment APE patients). The collected serum samples were stored at −80°C until metabolomic analysis was performed. It took approximately 6 months from the collection of the blood sample to the metabolomic analysis.

### Metabolomics assays

The preparation of serum samples: After thawing, the serum sample was mixed with cold methanol in a ratio of 1:3 to precipitate protein and extract metabolites. 200 μL serum sample of each subject was placed in the 1.5 mL EP tube, and 600 μL methanol was added. After vortex (Vortex oscillator, Scientific industries, United States) and ultrasonic (Ultrasonic cleaner, Kunshan Ultrasonic Instruments Company, Kunshan) for 15 min, the mixture was centrifuged at the speed of 1,500 r/min at 4°C for 15 min (Low-temperature high-speed centrifuge, Eppendorf, Germany). Next, the liquid supernatant was transferred to the new EP tube and repeated centrifuge twice. And then, the supernatant was filtered through a 0.22 μm needle filter (Tapery, Nanjing). The quality control (QC) sample was obtained by mixing an equal volume of the prepared sample.

The metabolomics was conducted by ultra-high-performance liquid chromatography–tandem mass spectrometry (UPLC–MS/MS) systems (AB Sciex, United States) and using ACQUITY UPLC® BEH C18 chromatography (Waters, Shanghai). The analysis was performed on the C18 chromatographic column (1.7 μm, 2.15 mm column) with the mobile phase comprising acetonitrile-water (containing 0.1% formic acid) flowing at 0.3 mL/min and injecting 5 μL at 40°C column temperature in a gradient elution manner. The process of gradient elution manner was detailed as follows: 0 ~ 2.0 min, acetonitrile was 5 ~ 20%; 2.0 ~ 4.0 min, acetonitrile was 20 ~ 25%; 4.0 ~ 9.0 min, acetonitrile was 25 ~ 60%; 9.0 ~ 14.0 min, acetonitrile was 60 ~ 100%; 14.0 ~ 18.0 min, acetonitrile was 100%; 18.0 ~ 18.1 min, acetonitrile was 100 ~ 5%; 18.1 ~ 19.5 min, acetonitrile was 5%. Atmospheric pressure chemical ionization sources adopted positive ion and negative ion scanning modes, respectively, to acquire the sample mass spectrum signal acquisition. For eliminated the analysis noise, ultrapure water as the blank sample was detected three times before each analysis batch. Ultrapure water was obtained by the Milli-Q Advantage A10 Ultrapure water system (Millipore, United States). Samples are analyzed randomly to avoid systematic errors caused by instrument signal fluctuations. Moreover, a QC sample was set for every five samples during the batch. Throughout the analysis, samples were placed in the automatic sampler at 4°C. And the solvents, methanol (Merck, Germany), acetonitrile (Merck, Germany), and formic acid (Thermo Fisher, United States), were all LC–MS grade.

### Statistical analysis

Data analysis was performed by SIMCA-P 14.0, and R. Data are expressed as mean ± standard deviation. When comparing differences in clinical variables, we used the Kruskal-Wallis test for continuous variables and the chi-square test or Fisher’s exact test for categorical variables. SIMCA-P 14.0 was used to subject the metabolomic data to multivariate analysis in different models. Serum metabolomic data from each group were preprocessed by unit variance scaling (UV Scaling) and mean centering. After preprocessing, model analysis was performed using orthogonal partial least squares projection to latent structure-discriminant analysis (OPLS-DA). Univariate analyses consisting of t-tests and fold change (FC) analyses were performed on the different variables, and volcano plots were drawn. FC is the ratio of metabolite levels in patients with APE and controls. Receiver operating characteristic (ROC) curves were used to assess the performance of metabolites in diagnosing APE. We finally used the criteria of *p*-value<0.05 and FC > 1.5/<0.67 to select differential metabolites. The LASSO regression algorithm was used for biomarker discovery, and logistic regression was used to construct diagnostic models of disease and to assess model performance based on ROC curves.

## Results

### Clinical and demographic characteristics of the study cohort

**Figure 1:**
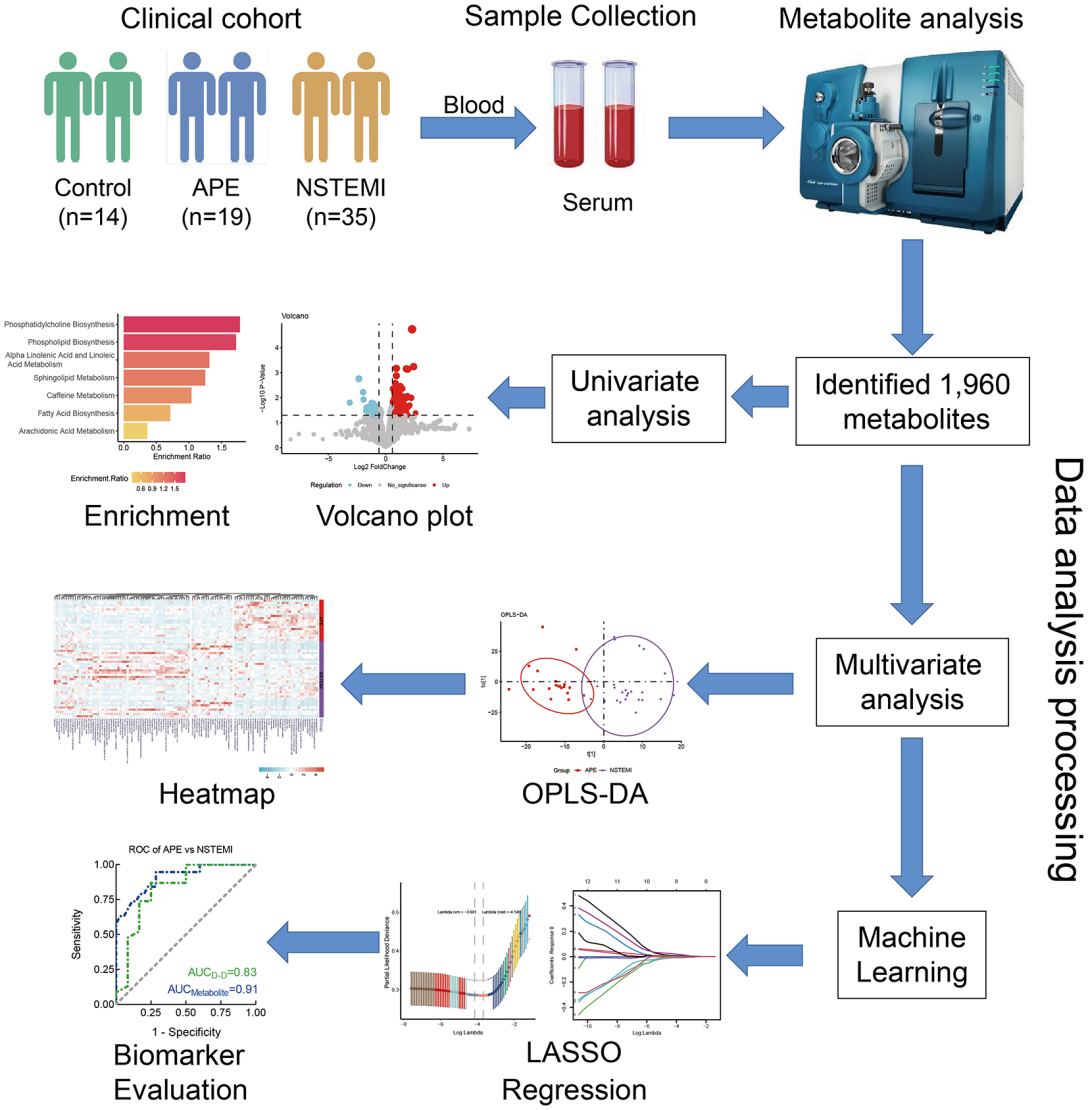
Study design and workflow of biomarker discovery. APE, acute pulmonary embolism; NSTEMI, non-ST segment elevation myocardial infarction; OPLS- DA, orthogonal partial least squares discriminant analysis; LASSO, least absolute shrinkage, and selection operator; ROC, receiver operating characteristic.

The whole study was designed with a concise workflow as shown in Figure 1. A total of 68 serum samples were collected from subjects in this study, of which 19 were from patients with APE, 35 were from patients with NSTEMI, and 14 were from healthy individuals for analysis. The clinical characteristics of APE, NSTEMI patients, and healthy subjects are shown in Table 1. Patients were all over 60 years of age and did not differ significantly from healthy individuals (*p*-value>0.05). Other indicators, such as gender, BMI, smoking, and alcohol consumption, were not significantly different between the APE patients and the other groups (*p*-value>0.05). Yet, the concentration of D-dimer in the blood of APE patients was significantly higher than that of NSTEMI patients (*p*-value<0.01) and healthy individuals (*p*-value<0.001) (Supplementary Figure S1). Significant changes in the blood of patients with APE, such as B. type brain natriuretic peptide precursors, white blood cells, neutrophils, and lymphocytes, indicate the severity of the disease and may pose a threat to the patient’s health (Supplementary Table S1).

**Table 1:** Clinical characteristics of the subjects.

Characteristics	APE	NSTEMI	Health	*p*-value
	(*n* = 19)	(*n* = 35)	(*n* = 14)	
Age (year)	69.0 (64.0, 77.0)	66.5 (57.3, 72.3)	58.0 (57.0, 71.3)	0.150
Sex				
Female	8 (42.1%)	12 (34.3%)	5 (35.7%)	0.847
Male	11 (57.9%)	23 (65.7%)	9 (64.3%)	
BMI (kg/m^2^)	23.7 (22.6, 27.6)	25.0 (23.1, 28.7)	25.6 (23.1, 27.3)	0.722
Smoking				
No	11 (57.9%)	14 (40.0%)	8 (57.1%)	0.350
Yes	8 (42.1%)	21 (60.0%)	6 (42.9%)	
Drinking				
No	10 (52.6%)	13 (37.1%)	9 (64.3%)	0.193
Yes	9 (47.4%)	22 (62.9%)	5 (35.7%)	
D-dimer (μg·mL^−1^)	0.8 (0.5, 3.1)	0.3 (0.2, 0.4)	0.1 (0.1, 0.2)	<0.001
COPD				
No	17 (89.5%)	34 (97.1%)	14 (100.0%)	0.282
Yes	2 (10.5%)	1 (2.9%)	0 (0%)	
Pneumonia				
No	16 (84.2%)	35 (100.0%)	14 (100.0%)	0.017
Yes	3 (15.8%)	0 (0%)	0 (0%)	
Coronary disease				
No	13 (68.4%)	25 (71.4%)	14 (100.0%)	0.064
Yes	6 (31.6%)	10 (28.6%)	0 (0%)	
Cerebral infarction				
No	17 (89.5%)	31 (88.6%)	14 (100.0%)	0.423
Yes	2 (10.5%)	4 (11.4%)	0 (0%)	
Hypertension				
No	9 (47.4%)	18 (51.4%)	14 (100.0%)	0.003
Yes	10 (52.6%)	17 (48.6%)	0 (0%)	
Diabetes				
No	16 (84.2%)	23 (65.7%)	14 (100.0%)	0.024
Yes	3 (15.8%)	12 (34.3%)	0 (0%)	
Hyperlipidemia				
No	14 (73.7%)	27 (77.1%)	14 (100.0%)	0.119
Yes	5 (26.3%)	8 (22.9%)	0 (0%)	
Previous myocardial infarction				
No	19 (100.0%)	29 (82.9%)	14 (100.0%)	0.045
Yes	0 (0%)	6 (17.1%)	0 (0%)	

Hypertension (high blood pressure) is when the pressure in your blood vessels is too high (140/90 mmHg or higher). Data are *n* (%), median (p25-p75).

### Comparison of serum metabolic profiling between the APE and healthy groups

**Figure 2:**
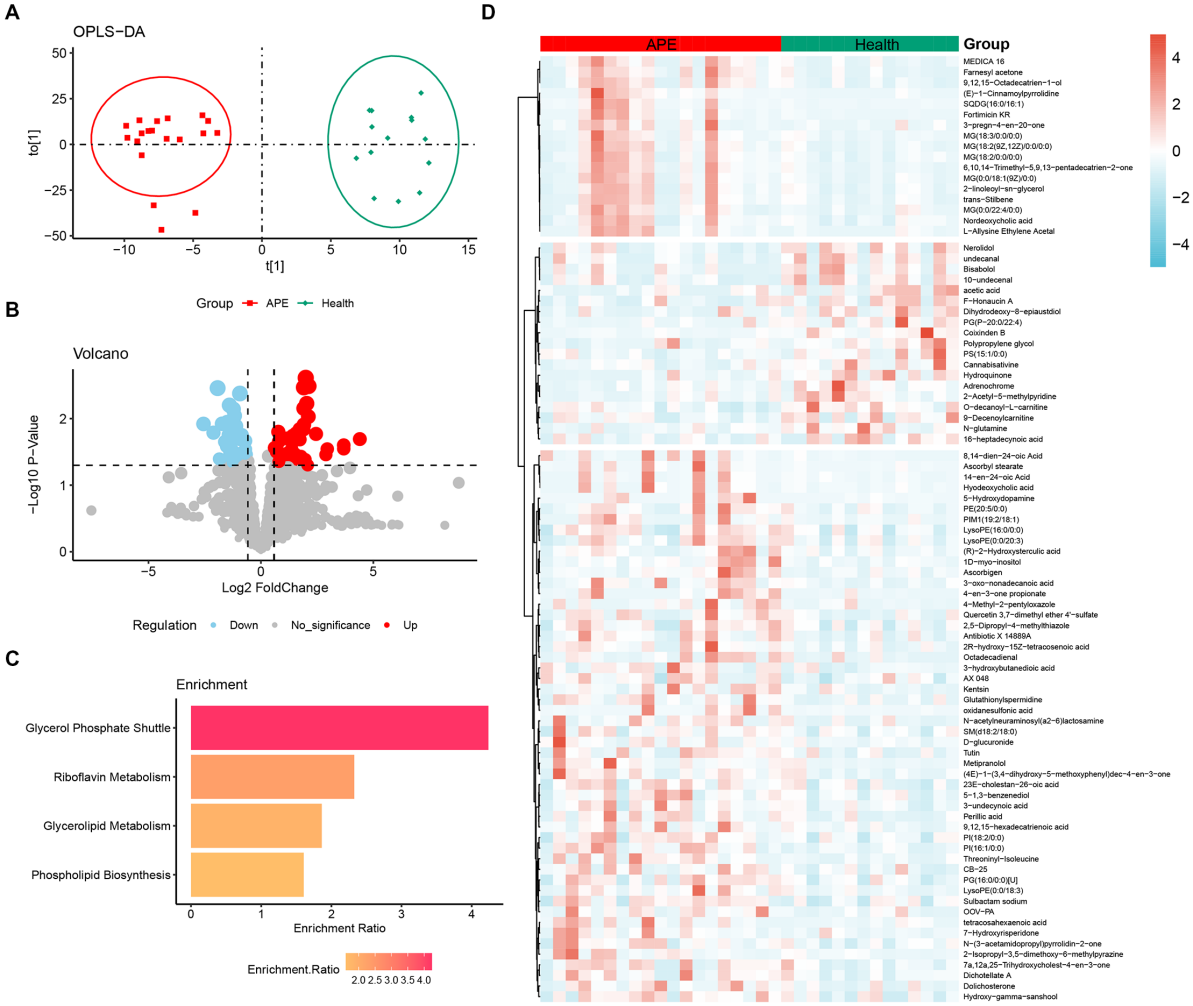
Analysis of metabolomic differences between APE patients and healthy controls. **(A)** OPLS-DA score plot between APE patients and healthy controls; **(B)** Volcano plot between APE patients and healthy controls. Red dots represent metabolites with increased concentrations, blue dots represent metabolites with decreased concentrations, and grey dots represent metabolites with no significant change in concentration in the APE. **(C)** Pathway analysis of differential metabolites between APE patients and healthy controls; **(D)** Heatmap of differential metabolites between APE patients and healthy controls.

In this study, a total of 1960 metabolites were detected. These metabolites can be classified into 17 categories, with the most represented being glycerophospholipids (17%) and fatty acyl (17%), followed by propanolipids (7%) (Supplementary Figure S2). First, to identify metabolites associated with the occurrence of APE, we focused on the differential metabolites between APE and controls. Using the OPLS-DA model, classification results showed a significant separation of all serum metabolites between APE and controls (Figure 2A). Next, we screened for components of variation based on fold change (FC) and *p*-values (FC > 1.5/<0.67 and *p*-values <0.05). The results of differential metabolite filtering are shown in the volcano plot (Figures 2B,D). A total of 88 differential metabolites were identified, including 19 down-regulated and 69 up-regulated differential metabolites. Finally, we performed a KEGG pathway enrichment analysis of serum differential metabolites between APE patients and healthy individuals to further analyze the metabolomics data. 88 differential metabolites were enriched in four major metabolic pathways including glycerophosphate shuttle, riboflavin metabolism, glycerolipid metabolism, and phospholipid biosynthesis (Figure 2C).

### Comparison of serum metabolic profiling between the APE and NSTEMI groups

**Figure 3:**
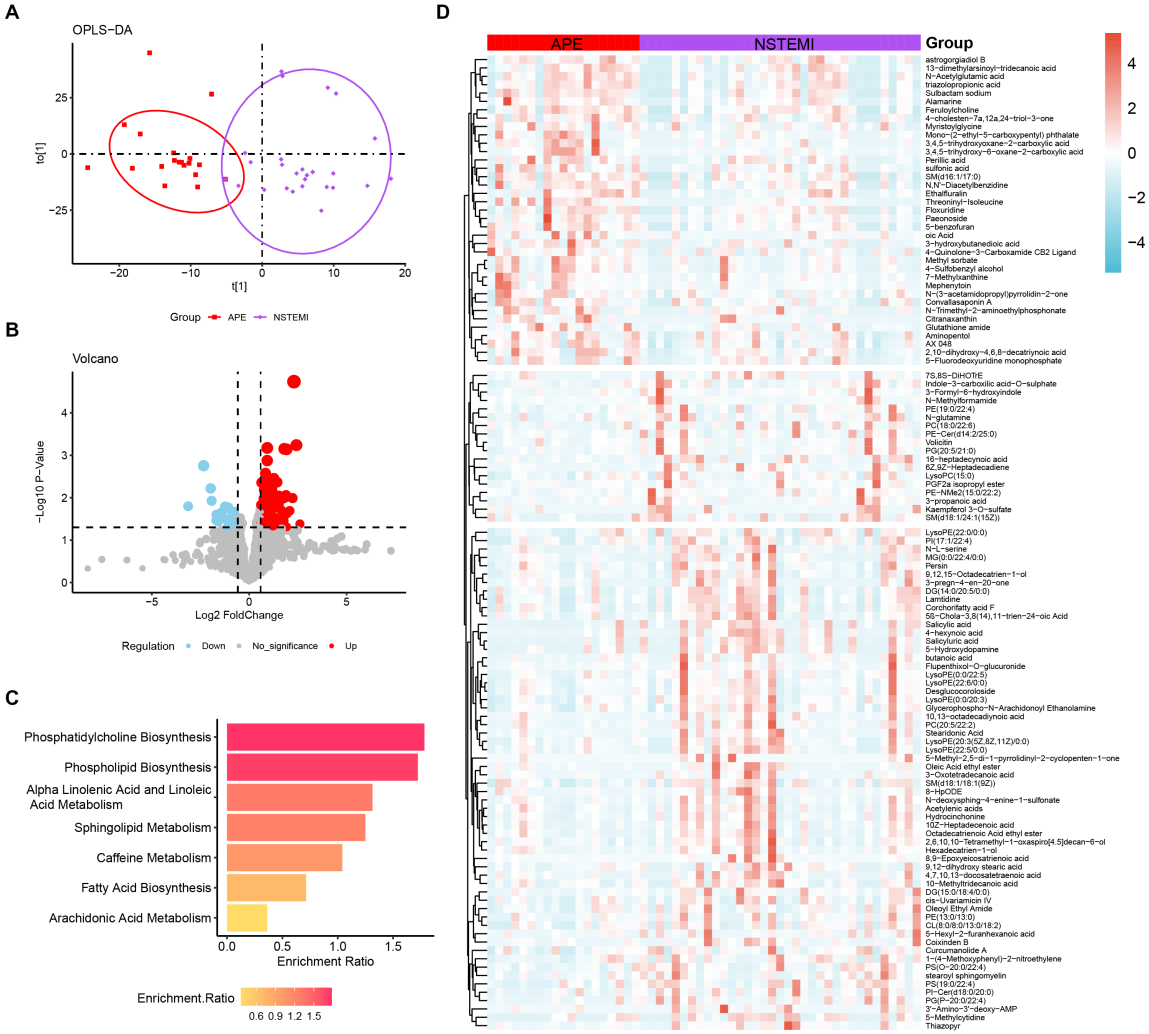
Analysis of metabolomic differences between APE patients and NSTEMI patients. **(A)** OPLS-DA score plot between APE and NSTEMI patients; **(B)** Volcano plot between APE and NSTEMI patients. Red dots represent metabolites with increased concentrations, blue dots represent metabolites with decreased concentrations, and grey dots represent metabolites with no significant change in concentration in the APE. **(C)** Pathway analysis of differential metabolites between APE and NSTEMI patients; **(D)** Heatmap of differential metabolites between APE and NSTEMI patients.

In clinical practice, the similarity of symptoms between NSTEMI and APE is not conducive to timely and appropriate treatment. Therefore, accurate differentiation between NSTEMI and APE is necessary. The OPLS-DA score plot shows that patients with APE are significantly different from those with NSTEMI (Figure 3A). A total of 115 different metabolites were identified, including 37 up-regulated and 78 down-regulated (Figures 3B,D). Enrichment analysis of the KEGG pathway for the differential metabolites between the two groups showed that the pathways represented by phospholipid biosynthesis, phosphatidylcholine biosynthesis, and sphingolipid metabolism were disrupted (Figure 3C).

### Machine learning-based biomarker panel selection for classification of APE, NSTEMI, and healthy individuals

**Figure 4:**
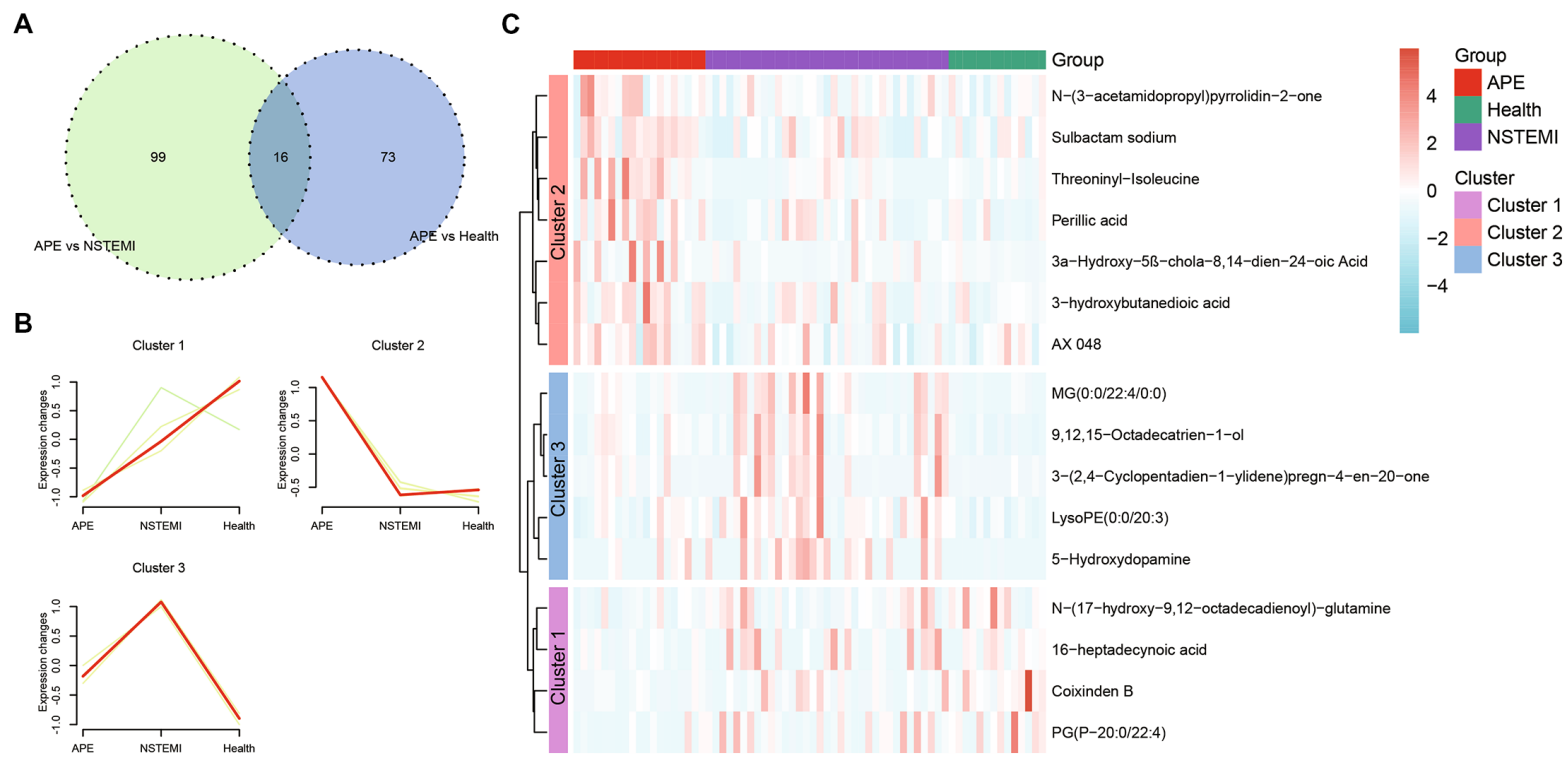
Shared differential metabolites between APE patients, NSTEMI patients, or healthy controls. **(A)** Venn diagram of differential metabolites between APE patients, NSTEMI patients, or healthy controls; **(B)** Clustering diagram of differential metabolites between APE patients, NSTEMI patients, or healthy controls; **(C)** Heatmap of differential metabolites between APE patients, NSTEMI patients, or healthy controls.

**Figure 5:**
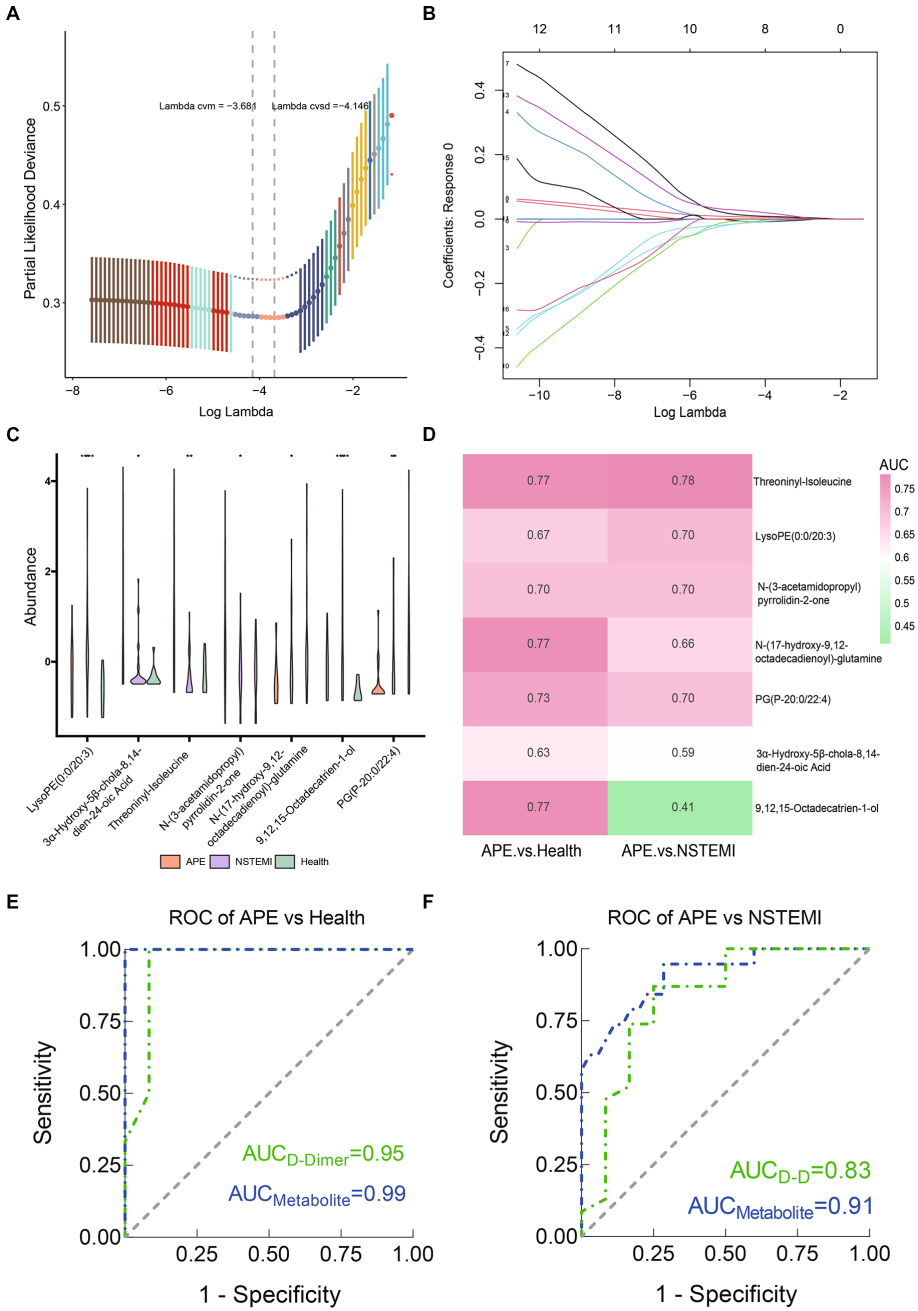
Screening of biomarkers and constructing and evaluating diagnostic models between APE patients, NSTEMI patients, or healthy controls. **(A)** Selection of the optimal parameter in the least absolute shrinkage and selection operator (LASSO) regression with tenfold cross-validation; **(B)** LASSO coefficient profiles of the candidate metabolites. The vertical line is drawn at the value selected using 10-fold cross-validation in log (λ) sequence and showed ten coefficients with non-zero were indicated. **(C)** Seven screened metabolites violin diagram; **(D)** Heatmap of the AUC values of the seven screened metabolites; **(E)** ROC curves of metabolite models and D-Dimer between APE patients and healthy controls; **(F)** ROC curves of metabolite models and D-Dimer between APE patients and NSTEMI patients.

As described above, we identified 88 differential metabolites between patients with APE and healthy individuals, and 115 differential metabolites between patients with APE and patients with NSTEMI. To assess the impact of these metabolites on the diagnosis of APE patients, NSTEMI patients, and healthy individuals, we used 16 shared differential metabolites for follow-up analysis (Figure 4A). For these 16 metabolites, the trends in distribution between groups were grouped into three categories (Figures 4B,C). Based on the selection of the LASSO regression algorithm, we obtained seven candidate metabolites (Figures 5A–C). The area under the ROC curve (AUC) for these metabolites mainly ranged from 0.6 to 0.8, showing the good diagnostic performance (Figure 5D). To further improve the AUC, a logistic regression method was used to construct a diagnostic model. The results showed that the metabolite model for APE patients and healthy individuals had an AUC value of 0.99, which was significantly higher than that for D-dimers (AUC = 0.95); the metabolite model for APE patients and NSTEMI patients had an AUC value of 0.91, which was considerably higher than that for D-dimers (AUC = 0.83) (Figures 5E,F). This demonstrates the high diagnostic performance of the metabolite model.

## Discussion

To systematically identify the metabolic profile and related pathways of APE development, we carried out a non-targeted metabolomics investigation. Metabolomic profiling revealed significantly different pathways in patients with APE compared to control participants, with the most significant alterations in glycerophosphate shuttle, and riboflavin metabolism. In addition, we developed a comprehensive biomarker discovery strategy to further select a combination of biomarkers based on seven metabolites, which has potential clinical utility for the classification of APE using two machine learning algorithms (LASSO and logistic regression) and is an effective method for assessing and predicting disease status. The plasma biomarker panel distinguished APE from controls with significantly high performance and an AUC of over 0.9. More importantly, we developed a predictive model based on seven metabolites to distinguish APE from NSTEMI with high validity. Our findings highlight the role of specific metabolites as novel early diagnostic and risk biomarkers for APE. Our selected metabolites have excellent accuracy and predictive power, and their combination may be useful for the diagnosis of APE.

Due to its atypical clinical picture, APE is a latent disease with a high mortality rate and diagnosis becomes very difficult (17). Finding meaningful and differential metabolites through metabolomic analysis techniques can help diagnose APE. Metabolites are sensitive and subtle, so it is possible that they could be a potential biomarker for early diagnosis of APE. By complementing diagnostic biomarkers, it can be used to assist in the pathological staging of APE and subsequent precise treatment decisions. Since the study aimed to improve the diagnostic of APE analizing and validating a new tool for early diagnosis. However, there is no statistical analysis developed and serial samples collected to assess and to monitor the evaluation in disease course.

When comparing patients with APE and healthy controls, the increased WBC and ANC could be inflammation, which plays an important role in thrombosis. It can cause damage to the vascular endothelium, release procoagulants, activate coagulation, and ultimately lead to thrombosis. Moreover, thrombus can also stimulate inflammation, leading to platelet release, aggregation, and adhesion of leukocytes, such as neutrophils, which is an important marker of inflammation (18, 19). D-D is a degradation product of cross-linked fibrin, reflecting an active hypercoagulable and secondary fibrinolytic state. When APE occurs, D-D is always elevated, so it is used to exclude non-APE patients.

For differential metabolites between patients with APE and healthy controls, the main pathways are phosphate-related pathways involving energy metabolic processes such as glycolysis, including the metabolism of D-glucose, which can be converted to glyceric acid and deoxyribose via the pentose phosphate pathway, with elevated glyceric acid and decreased deoxyribose. An increase in the pentose phosphate pathway is accompanied by a decrease in the citric acid cycle, which may lead to an accumulation of pyruvate. Clinical examination shows that patients with APE have higher LDH levels than healthy controls. Under hypoxic conditions, pyruvate produced by glycolysis can be reduced to lactate by NADH under LDH. increased LDH may indicate that more pyruvate is presently requiring a reaction. Hydroquinone levels are reduced in patients with APE. Homocysteine (Hcy) is an amino acid containing sulfhydryl groups and is an intermediate metabolite of methionine (20). Clinical examinations have found that Hcy levels are much higher in patients with APE than in healthy individuals. Many studies have also found that Hcy is elevated in APE and is a risk factor for APE. Hcy and its derivatives promote platelet production of thromboxane, which promotes clotting and inhibits anticoagulation, thereby promoting thrombosis (21–23). More importantly, in the metabolism of cysteine and methionine, Hcy can be methylated to produce methionine or cysteine via the transsulfuration pathway. Cysteine and methionine deficiency are usually accompanied by hyperhomocysteinemia.

The results show that (S)-3-sulfolactate is decreased in the metabolism of cysteine and methionine. Disulfide bonds formed by Hcy are essential for maintaining the spatial structure of proteins and the activity of many important enzymes, such as succinate dehydrogenase and lactate dehydrogenase, which may also affect the accumulation of metabolites of glycolysis and intermediates of the citric acid cycle, as other previous studies have concluded (13). The reasons for the changes in these metabolites and the involvement of these pathways may be related to the characteristics of APE pathogenesis. During an APE episode, the thrombus breaks away from the blood vessels and enters the right ventricle via the venous system, which then obstructs the pulmonary arteries and branches, causing increased pulmonary vascular resistance, increased pulmonary artery pressure, and inadequate oxygen supply. Normally, glucose is converted to pyruvate and acetyl-CoA by glycolysis, and acetyl-CoA then participates in the citric acid cycle. Under adequate oxygen, acetyl-CoA reacts with reducing coenzyme groups to form ATP by oxidative phosphorylation in the mitochondria, whereas under hypoxic conditions, glucose will be consumed more by glycolysis and the pentose phosphate pathway and less by the aerobic oxidative pathway, the citric acid cycle. Due to hypoxia, the rate of oxidative phosphorylation is low and intermediate products of the citrate cycle accumulate.

The comparison of differential metabolites between APE patients and NSTEMI patients involved 115 differential metabolites, 37 of which were up-regulated and 78 down-regulated. Pulmonary surfactant (PS) consists of phospholipids and proteins and plays a crucial role in alveolar function (24, 25). Phospholipids make up more than 90% of the PS and include phosphatidylcholine (PC), phosphatidylglycerol (PG), sphingomyelin (SPH), phosphatidylinositol (PI) and phosphatidylethanolamine, of which the major component is PC (26). When APE strikes, increased release of inflammatory factors and activation of phosphatases, among others, are together involved in the development and progression of lung injury. Phosphatases are involved in many biological responses such as inflammation, phosphorylation, and the initiation of arachidonic acid metabolism. Activation of phosphatases has a clear and direct effect on lung injury. It uses PC and other phospholipids as substrates and degrades fatty acids at the sn-2 position, producing lysophospholipids and free fatty acids, leading to a decrease in PC levels and thus affecting alveolar function. In addition, phosphatidylethanolamine produced from CDP-ethanolamine and diglycerides can also be converted to PC by reacting with S-adenosylmethionine in the presence of phosphatidylethanolamine methyltransferase. A decrease in CDP-ethanolamine may reduce PC levels. Tryptophan is metabolized *in vivo* mainly via the canine urinary quinoline pathway or the 5-hydroxytryptamine pathway, and 2-aminobenzoic acid is one of the metabolites of the canine urinary quinoline pathway (27). The high levels of pro-inflammatory factors released during inflammation can activate the kynurenine pathway and increase the metabolites of the kynurenine pathway. S1P was found to reduce neutrophil chemotaxis, convert the pro-inflammatory effects of macrophages into anti-inflammatory effects, and improve the inflammatory response (28, 29). Increased SIP in patients with APE may reduce APE-induced lung inflammation and promote recovery.

Clinically, the symptoms of APE and NSTEMI are very similar. The main symptoms of both diseases include chest discomfort, breathlessness, and nausea, making it difficult to distinguish between the two. In clinical practice, the earlier the disease is identified, the more appropriate treatment can be administered, improving prognosis and reducing mortality. To differentiate between APE and NSTEMI, it is necessary to study the different metabolites between the two diseases. The different metabolites between patients with APE and NSTEMI during an attack are mainly involved in the biosynthesis of phospholipids and phosphatidylcholine, and the metabolism of arachidonic acid. Thromboxane B2 (TXB2) and 12-HETE are higher in patients with APE than in patients with NSTEMI. 12-HETE is a metabolite of arachidonic acid. It promotes an increase in inflammatory mediators and vascular permeability (30). When platelets are activated, arachidonic acid is released by activated phospholipase A2, catalyzed by 12-lipoxygenase, which then produces 12-HETE (31–33).

For the metabolic markers screened, they are similarly involved in the complex metabolic activities of the disease. Threoninyl-Isoleucine, a dipeptide composed of threonine and isoleucine, is a transient intermediate in protein hydrolysis into a specific amino acid degradation pathway that is significantly altered in patients with colorectal cancer (34). LysoPE (0:0/20:3) is a lysophosphatidylethanolamine or lysophospholipid that plays a role in signaling, and further observations are needed regarding the role it plays in APE attacks. N-(3-acetamidopropyl) pyrrolidin-2-one, a catabolic product of spermidine, is formed from N1 acetyl spermidine and has been found at elevated levels in the urine of patients with non-Hodgkin’s lymphoma (35). The implications of the significant changes in APE for these metabolites warrant subsequent in-depth investigation.

### Limitations

This study’s potential limitations include, firstly, the relatively small number of patients and thus the need for a large multicentre clinical to conduct the trial to assess the accuracy and robustness of the panel as a non-invasive biomarker for APE. Secondly, the study is hospital-based and may be confounded by selection bias. Thirdly, the differential metabolites observed between the study and control groups were based on non-targeted metabolomic techniques, and thus the reliability of the results needs to be further confirmed by targeted metabolic techniques. Furthermore, the detailed role of biomarker metabolites in the pathogenesis of APE needs further elucidation or experimental validation.

## Conclusion

In conclusion, our current study confirms the existence of aberrant serum metabolite variations between APE patients, NSTEMI patients, and healthy individuals, which may have important implications for our better understanding of the pathophysiology of the disease.

## Data availability statement

The original contributions presented in the study are included in the article/Supplementary material, further inquiries can be directed to the corresponding authors.

## Ethics statement

The studies involving human participants were reviewed and approved by Huabei Petroleum Administration General Hospital. The patients/participants provided their written informed consent to participate in this study.

## Author contributions

MX and RL: conception and design and drafting. YL, HZ, XG, and RL: acquisition of data. MX, YL, HZ, XG, and RL: analysis and interpretation of data and critical revision. All authors contributed to the article and approved the submitted version.

## Funding

This study was funded by the Key Research and Development Project of Hebei Province (grant number 182777198) and the National Natural Science Foundation of China grants (grant numbers 81872579 and 82173479).

## Conflict of interest

The authors declare that the research was conducted in the absence of any commercial or financial relationships that could be construed as a potential conflict of interest.

## Publisher’s note

All claims expressed in this article are solely those of the authors and do not necessarily represent those of their affiliated organizations, or those of the publisher, the editors and the reviewers. Any product that may be evaluated in this article, or claim that may be made by its manufacturer, is not guaranteed or endorsed by the publisher.
