# Supplementary material for: Serum metabolic signatures for acute pulmonary embolism identified by untargeted metabolomics
Source: Front Med (Lausanne). 2023 Jun 2;10:1169038. doi: 10.3389/fmed.2023.1169038 (PMC10272531; doi:10.3389/fmed.2023.1169038)
Supplement: Supplementary file 1 [file Data_Sheet_1.PDF]

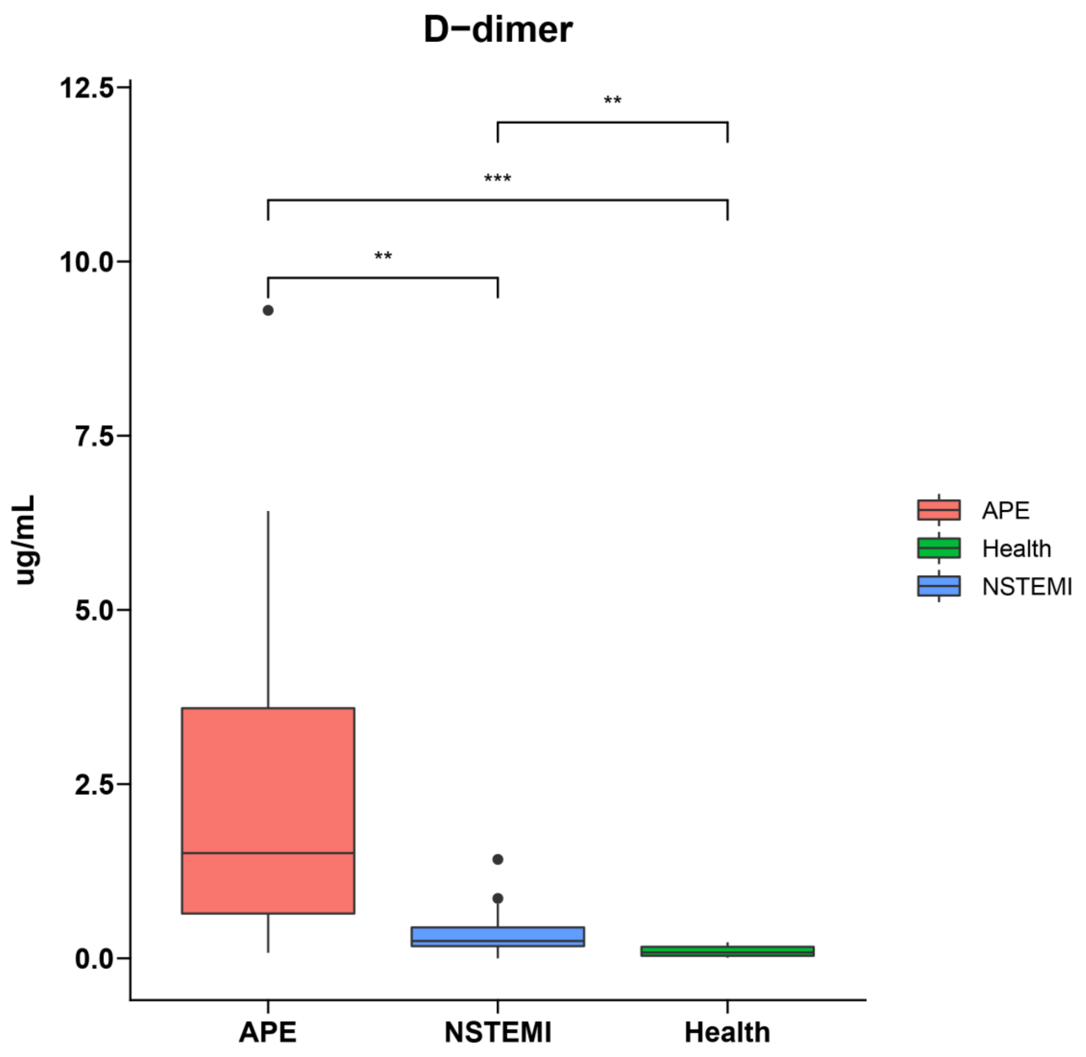

FIGURE S1 Distribution of D-dimers among APE patients, NSTEMI patients, and healthy controls. **p value< 0.01, ***p value< 0.001.

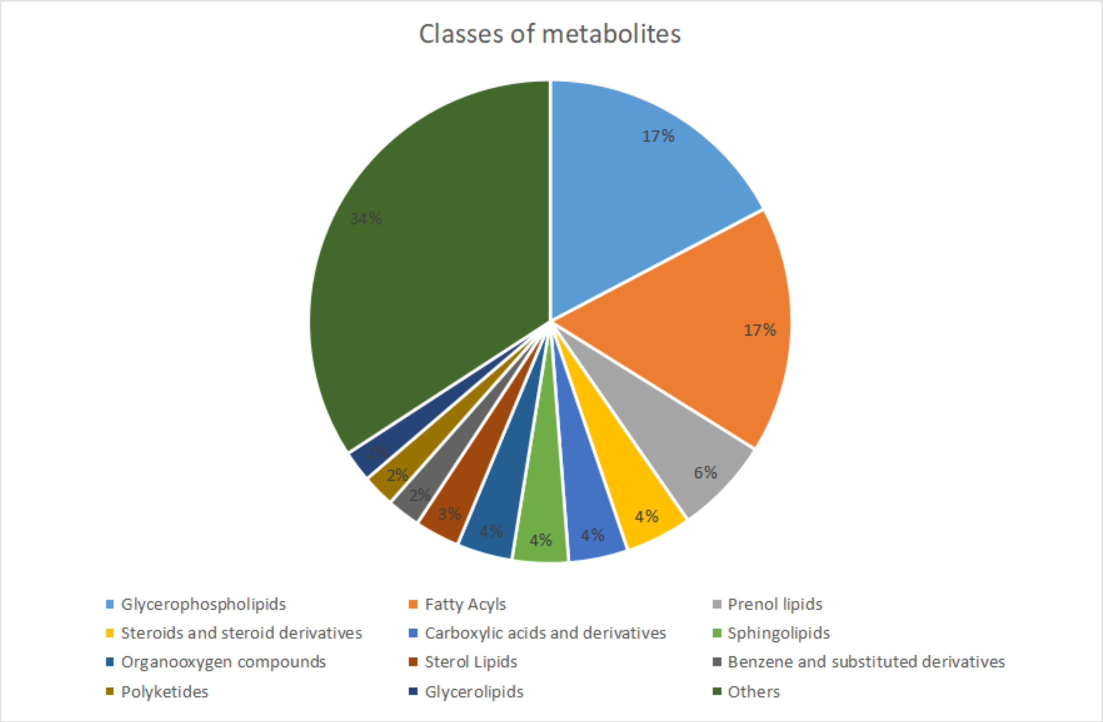

FIGURE S2 Classification of metabolites in the ser of APE patients, NSTEMI patients, and healthy controls.

TABLE S1 Results of blood biochemistry in subjects.

Characteristics	APE (N=19)	NSTEMI (N=35)	Health (N=14)	P-value
**Prothrombin time (s)**	12.0 (11.4-13.7)	11.6 (10.8-12.8)	10.6 (9.8-11.5)	<0.001
**Prothrombin activity degree (%)**	93.0 (70.6-100.0)	100.3 (89.4-113.3)	101.0 (91.8-111.3)	0.069
**International Normalised Ratio (INR)**	1.1 (1.0-1.3)	1.0 (1.0-1.1)	1.0 (0.9-1.0)	<0.001
**Activated partial thromboplastin time (s)**	30.2 (27.7-31.9)	30.0 (26.3-32.5)	31.5 (28.2-33.3)	0.532
**Fibrinogen (g/L)**	3.2 (2.3-4.2)	2.8 (2.6-3.3)	4.1 (3.0-4.5)	<0.001
**Prothrombin time measurement (s)**	15.1(13.8-16.4)	15.0 (14.3-16.6)	13.4 (11.7-15.0)	<0.001
**Myoglobin (ng/ml)**	26.2(16.1-74.1)	44.3 (27.2-107.6)	43.3 (25.8-75.1)	0.157
**Troponin (ng/ml)**	0.04 (0.02-0.6)	1.0 (0.3-2.6)	0.06 (0.03-0.08)	<0.001
**B-type brain natriuretic peptide precursor (pg/ml)**	570.2 (162.0-1260.0)	390.7 (209.4-1075.0)	261.6 (128.1-390.2)	0.032
**Leukocytes (* 10^9/L)**	7.2 (6.3-10.0)	8.6 (6.3-10.2)	5.2 (4.6-5.9)	<0.001
**Neutrophil percentage (%)**	73.4 (53.0-80.4)	67.3 (63.6-74.0)	54.5 (49.0-62.8)	<0.001
**Lymphocytes percentage (%)**	14.6 (10.7-31.9)	24.1 (19.3-28.0)	35.4 (28.5-40.5)	<0.001
**Monocytes percentage (%)**	7.2 (5.8-8.4)	5.4 (4.6-7.6)	6.8 (5.9-7.9)	0.019
**Eosinophils percentage (%)**	1.2 (0.3-3.6)	1.1 (0.6-1.7)	1.7 (1.2-2.6)	0.022
**Basophils percentage (%)**	0.4 (0.2-0.7)	0.3 (0.2-0.4)	0.4 (0.3-0.7)	0.091
**Absolute Neutrophil (* 10^9/L)**	5.2 (3.2-7.9)	5.7 (4.5-6.9)	2.8 (2.4-3.6)	<0.001
**Absolute lymphocytes (* 10^9/L)**	1.4 (1.0-1.8)	1.8 (1.5-2.2)	1.8 (1.5-1.9)	0.071
**Absolute monocyte value (* 10^9/L)**	0.6 (0.4-0.8)	0.4 (0.3-0.7)	0.4 (0.3-0.4)	0.007
**Absolute eosinophils (*10^9/L)**	0.07 (0.03-0.2)	0.09 (0.06-0.1)	0.09 (0.07-0.1)	0.742
**Absolute basophils (* 10^9/L)**	0.03 (0.02-0.05)	0.02 (0.02-0.03)	0.02 (0.02-0.03)	0.220
**Haemoglobin (g/L)**	131.0 (120.3-138.5)	142.0 (129.8-149.3)	143.0 (134.3-151.0)	0.017
**Erythrocytes (* 10^{12}/L)**	4.2 (3.9-4.7)	4.6 (4.1-4.9)	4.6 (4.4-4.9)	0.221
**Erythrocyte specific volume (%)**	39.1 (35.6-42.0)	42.0 (38.3-44.3)	41.7 (39.7-43.7)	0.088

**Mean red blood cell volume (fL)**	90.0 (87.4-95.5)	91.1 (88.5-94.7)	91.0 (88.3-93.1)	0.936
**Mean erythrocyte haemoglobin volume (pg)**	29.8 (29.1-32.4)	30.7 (30.3-31.9)	31.1 (29.9-31.9)	0.330
**Mean red blood cell haemoglobin concentration (g/L)**	332.0 (321.5-335.8)	343.0 (333.0-348.5)	341.0 (335.8-345.3)	0.014
**Erythrocyte distribution width (CV) (%)**	13.6 (12.7-14.0)	12.9 (12.2-13.1)	12.7 (12.3-13.0)	0.040
**Erythrocyte Distribution Width (SD) (fL)**	44.3 (41.2-48.2)	42.6 (40.9-45.6)	41.7 (40.6-43.9)	0.044
**Platelet count (*10^9/L)**	210.0 (154.0-243.0)	215.0 (172.5-239.0)	206.0 (176.8-247.5)	0.672
**Platelet Specific Volume (%)**	0.3 (0.2-0.3)	0.2 (0.2-0.3)	0.2 (0.2-0.3)	0.934
**Mean platelet volume (fL)**	10.9 (10.4-11.3)	10.1 (9.2-10.9)	10.9 (10.2-11.4)	0.026
**Platelet Distribution Width (fL)**	13.8 (11.8-16.5)	16.0 (11.9-16.2)	13.0 (11.5-14.0)	0.064
**Large Platelet Ratio (%)**	32.1 (28.0-35.8)	26.2 (20.1-33.0)	32.1 (25.5-36.5)	0.038
**Total bilirubin (μ mol/L)**	17.9 (9.7-23.7)	11.7 (9.2-16.8)	16.3 (14.3-19.1)	0.045
**Direct bilirubin (μ mol/L)**	5.9 (3.8-9.0)	3.2 (1.9-4.9)	4.4 (3.1-5.3)	0.011
**Indirect bilirubin (μ mol/L)**	10.9 (6.6-16.8)	8.5 (6.0-14.7)	10.6 (4.7-11.8)	0.472
**Alanine aminotransferase (U/L)**	14.8 (12.2-23.4)	21.9 (14.0-30.3)	20.5 (15.3-25.9)	0.096
**Aspartate aminotransferase (U/L)**	22.0 (15.7-25.4)	32.4 (18.8-61.7)	23.2 (19.3-25.5)	0.059
**Glutathione/glutamate**	1.1 (0.7-1.6)	1.3 (0.8-2.0)	0.9 (0.9-1.4)	0.491
**Gamma-glutamyltransferase (U/L)**	28.9 (16.3-51.1)	22.8 (16.9-32.9)	21.3 (14.3-30.6)	0.277
**Alkaline phosphatase (U/L)**	68.2 (63.8-96.5)	72.5 (64.4-84.3)	65.1 (55.3-79.2)	0.261
**Cholinesterase (U/L)**	5974.0 (4776.0-7630.0)	8366.0 (7513.0 - 9083.0)	8870.0 (7941.0-10101.0)	<0.001
**Total bile acids (μ mol/L)**	3.5 (2.0-8.0)	2.6 (1.7-7.4)	6.9 (4.2-9.6)	0.413
**Total protein (g/L)**	62.6 (58.1-67.0)	66.8 (61.3-73.4)	70.5 (69.6-75.8)	0.002
**Albumin (g/L)**	35.5 (32.4-39.0)	40.9 (37.7-44.6)	44.8 (41.8-47.3)	<0.001

**Globulin (g/L)**	27.6 (23.8-29.1)	25.3 (22.5-30.6)	27.5 (22.5-29.7)	0.818
**Leukocyte ratio**	1.4 (1.1-1.8)	1.6 (1.4-1.9)	1.8 (1.5-2.1)	0.011
**Lactate dehydrogenase (U/L)**	199.0 (144.2-294.1)	227.4 (169.7-392.5)	174.9 (162.6-202.1)	0.014
**Alpha-hydroxybutyrate dehydrogenase (U/L)**	165.8 (118.6-224.3)	193.0 (141.4-228.6)	131.8 (107.3-151.3)	<0.001
**Creatine kinase (U/L)**	61.3 (28.4-92.5)	134.4 (107.5-516.6)	122.5 (73.8-169.2)	<0.001
**Creatine kinase isoenzyme (U/L)**	12.9 (10.5-18.1)	20.6 (13.5-61.3)	11.9 (8.7-21.4)	<0.001
**Total cholesterol (mmol/L)**	3.9 (3.3-4.7)	4.9 (4.0-5.0)	5.4 (4.1-5.9)	0.006
**Triglycerides (mmol/L)**	1.2 (0.8-1.5)	1.6 (1.1-2.3)	1.5 (1.0-2.1)	0.129
**High-density lipoprotein cholesterol (mmol/L)**	1.1 (1.0-1.5)	1.2 (1.1-1.3)	1.4 (1.2-1.9)	0.002
**Low-density lipoprotein cholesterol (mmol/L)**	2.0 (1.3-3.0)	2.6 (2.2-3.0)	2.8 (2.3-3.5)	0.041
**Apolipoprotein A1 (g/L)**	1.0 (1.0-1.2)	1.2 (1.1-1.3)	1.3 (1.3-1.5)	<0.001
**Apolipoprotein B (g/L)**	0.8 (0.6-0.8)	0.9 (0.8-1.0)	1.0 (0.9-1.0)	0.007
**Glucose (mmol/L)**	5.2 (4.8-5.5)	7.1 (5.5-10.6)	5.5 (5.0-6.2)	0.001
**Urea (mmol/L)**	5.3 (4.1-6.6)	5.7 (4.7-6.8)	5.6 (4.7-6.1)	0.800
**Creatinine (μ mol/L)**	74.0 (61.4-92.6)	72.9 (57.3-83.7)	73.7 (65.1-80.3)	0.653
**Uric acid (μ mol/L)**	296.2 (240.6-371.5)	331.5 (277.5-397.6)	363.5 (327.3-438.2)	0.097
**Cystatin C (mg/L)**	1.1 (1.0-1.2)	0.9 (0.8-1.1)	0.9 (0.8-0.9)	0.023
**Homocysteine (μ mol/L)**	18.3 (14.0-21.8)	16.6 (13.6-24.2)	14.2 (9.9-15.7)	0.099

Note: Data are median (IQR).
